# 1408. Increasing Access to Medications for Opioid Use Disorder in Patients Hospitalized with Severe Injection Related Infections: A Single Center Experience

**DOI:** 10.1093/ofid/ofac492.1237

**Published:** 2022-12-15

**Authors:** Jack Keegan, Rebecca Bauer, William Peppard

**Affiliations:** Medical College of Wisconsin, Milwaukee, Wisconsin; Medical College of Wisconsin, Milwaukee, Wisconsin; Medical College of Wisconsin, Milwaukee, Wisconsin

## Abstract

**Background:**

Hospitalizations for severe injection related infections (SIRI) amongst persons who inject drugs (PWID) are often complicated by patient directed discharge (PDD), incomplete antibiotic treatment and 90-day readmission. Despite a strong association with improved clinical outcomes, medications for opioid use disorder (MOUD) remain significantly underutilized in this patient population. Beginning in January of 2022, our institution revised several key policies that had limited inpatient use of MOUD while also implementing a new order set to facilitate MOUD inductions. This study seeks to quantify the clinical impact of these interventions in PWID admitted with SIRI.

**Methods:**

We first performed a retrospective analysis of all PWID hospitalized with SIRI between January 2018 and December 2020 to establish baseline rates of PDD, antibiotic completion and 90-day readmission in patients who received MOUD compared to those who did not. Subsequent analyses will utilize a quasi-experimental design to compare the same clinical outcomes in patients admitted before and after institutional guideline revision and MOUD order set implementation.

**Results:**

A total of 97 patients were included in our baseline assessment, of which 21 (21.6%) received MOUD while hospitalized. Of patients receiving MOUD, 11 (52.4%) were new inductions. Use of MOUD was associated with significantly lower rates of PDD (5 [23.8%] MOUD patients vs 40 [53.3%] no MOUD patients; OR, 0.273 [95% CI, 0.091-0.823]) and significantly higher rates of antibiotic completion (17 [81.0%] MOUD patients vs 36 [48.0%] no MOUD patients; OR, 4.6 [95% CI, 1.41-14.98]).

**Conclusion:**

Our baseline data agrees with existing literature in finding MOUD to be an effective way of reducing PDD and increasing antibiotic completion in PWID admitted with SIRI. However, overall MOUD utilization was low, particularly after accounting for the effect of individuals prescribed MOUD prior to admission. Forthcoming data will assess the impact of institutional guideline revision and MOUD order set implementation on PDD, antibiotic completion and hospital readmission in PWID admitted with SIRI.

**Disclosures:**

**All Authors**: No reported disclosures.

